# Innovative strategies to optimise colorectal cancer immunotherapy through molecular mechanism insights

**DOI:** 10.3389/fimmu.2024.1509658

**Published:** 2024-12-09

**Authors:** Quanjun Lin, Zhiqiang Wang, Jue Wang, Ming Xu, Xinyi Zhang, Peng Sun, Yihang Yuan

**Affiliations:** ^1^ Department of General Surgery, Tongren Hospital, Shanghai Jiao Tong University School of Medicine, Shanghai, China; ^2^ Clinical Medical College, Southwest Medical University, Luzhou, China

**Keywords:** colorectal cancer (CRC), single-cell RNA sequencing (scRNA-seq), tumor cell subtypes, FXYD5+ Tumor Risk Score (FTRS), energy metabolism in cancer

## Abstract

**Background:**

Colorectal cancer (CRC) is a leading cause of cancer-related deaths globally. The heterogeneity of the tumor microenvironment significantly influences patient prognosis, while the diversity of tumor cells shapes its unique characteristics. A comprehensive analysis of the molecular profile of tumor cells is crucial for identifying novel molecular targets for drug sensitivity analysis and for uncovering the pathophysiological mechanisms underlying CRC.

**Methods:**

We utilized single-cell RNA sequencing technology to analyze 13 tissue samples from 4 CRC patients, identifying key cell types within the tumor microenvironment. Intercellular communication was assessed using CellChat, and a risk score model was developed based on eight prognostic genes to enhance patient stratification for immunotherapeutic approaches. Additionally, *in vitro* experiments were performed on *DLX2*, a gene strongly associated with poor prognosis, to validate its potential role as a therapeutic target in CRC progression.

**Results:**

Eight major cell types were identified across the tissue samples. Within the tumor cell population, seven distinct subtypes were recognized, with the C0 *FXYD5*+ tumor cells subtype being significantly linked to cancer progression and poor prognosis. CellChat analysis indicated extensive communication among tumor cells, fibroblasts, and immune cells, underscoring the complexity of the tumor microenvironment. The risk score model demonstrated high accuracy in predicting 1-, 3-, and 5-year survival rates in CRC patients. Enrichment analysis revealed that the C0 *FXYD5*+ tumor cell subtype exhibited increased energy metabolism, protein synthesis, and oxidative phosphorylation, contributing to its aggressive behavior. *In vitro* experiments confirmed *DLX2* as a critical gene associated with poor prognosis, suggesting its viability as a target for improving drug sensitivity.

**Conclusion:**

In summary, this study advances our understanding of CRC progression by identifying critical tumor subtypes, molecular pathways, and prognostic markers that can inform innovative strategies for predicting and enhancing drug sensitivity. These findings hold promise for optimizing immunotherapeutic approaches and developing new targeted therapies, ultimately aiming to improve patient outcomes in CRC.

## Graphical Abstract

Article Research Flowchart. Firstly, we downloaded the clinical data of 4 colorectal cancer patients from GEO. After a series of data processing, we obtained the single-cell atlas of colorectal cancer. After that, we took TCs as the study object to obtain their complete single-cell atlas and performed trajectory analysis, enrichment analysis, transcription factor analysis and communication network analysis in turn. In addition, we also performed bulk analysis for colorectal cancer, classified high or low FTRS groups, and analysed the related prognosis, and also verified it with *in vitro* experiments, and the obtained results were consistent with our study.

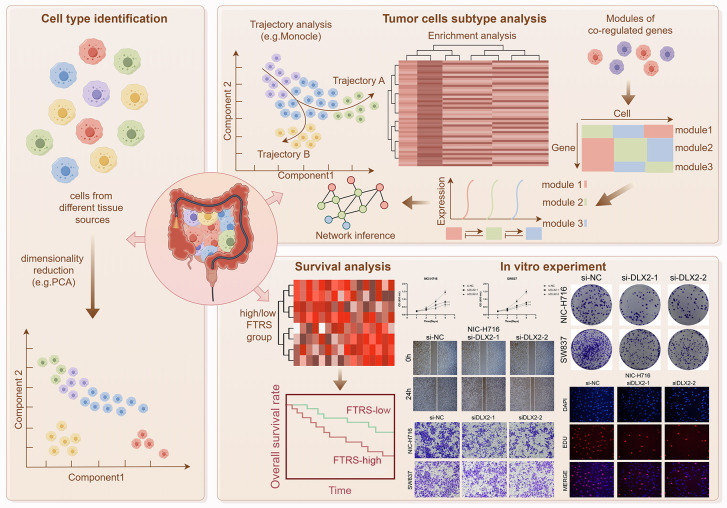

## 1 Introduction

CRC is one of the leading causes of cancer-related deaths globally and poses a major global health burden; it is the third most common malignant tumor and the second leading cause of cancer-related deaths worldwide (1, 2). It arises from the epithelial cells of the colon or rectum, often following a multi-step progression from normal tissue to adenoma (a benign tumor) and ultimately to carcinoma (a malignant tumor). This is a multi-step process that requires the accumulation of genetic/epigenetic aberrations (3–5). From a molecular point of view, CRC is caused by mutations in targeted oncogenes, tumor suppressor genes, and genes associated with DNA repair mechanisms. CRC originates from 3 main pathways: the adenoma-cancer sequence, the serrated pathway, and the inflammatory pathway (6). Depending on the origin of the mutation, colorectal cancer can be categorized as sporadic, hereditary, and familial (7, 8). Disease progression is influenced by a variety of genetic (9), environmental (10), and lifestyle factors, of which dietary factors are the most critical, shaping the gut microbiota to modulate regulation of colonic epithelial cell homeostasis and carcinogenesis, and preventing tumors in up to 50% of cases by improving lifestyle (7, 11–13). People with CRC usually present with breathlessness and fatigue. These are also common symptoms in patients with chronic heart failure (14–17). Currently, common treatments for CRC include a multimodal combination of surgery, radiotherapy and chemotherapy (18). However, recurrence and metastasis rates remain high. The combination of radiotherapy and immunotherapy may provide a new solution to this problem and has rapidly become the mainstay of treatment for many types of solid cancers, including CRC, but its future remains uncertain (19–22).

CRC is characterized by the heterogeneity of the tumor microenvironment (TME), with different types of cells playing different roles at different stages of tumorigenesis (23, 24). A comprehensive molecular characterization of human colon and rectal cancers has been previously revealed by deep whole genome sequencing (25). And as analytical tools continue to evolve, there are now a large number of studies that map individual cells in the TME at high resolution by scRNA-seq (26–28) and allow for clinical stratification of CRC (29). These analyses have revealed key cellular players, including epithelial cells, fibroblasts, immune cells such as T and B lymphocytes and tumor-associated macrophages. The latest emerging analytical technique, spatial transcriptomics analysis, has also been applied in CRC, helping to further advance targeted immunotherapy (30, 31).

In recent years, research has also focused on understanding the molecular drivers of CRC. Tumor cells (TCs) undergo genetic mutations that affect key signaling pathways, such as Wnt, Notch, and p53, which regulate cell growth, differentiation, and apoptosis (32–34). Furthermore, studies have shown that the epithelial-to-mesenchymal transition (EMT) process and the stemness properties of certain tumor subtypes play critical roles in CRC metastasis and recurrence (35, 36). Despite advancements in early detection and treatment (37), the prognosis for metastatic CRC remains poor (38, 39). Therefore, continued research into the molecular and cellular mechanisms driving CRC is essential for developing more effective therapies and improving patient outcomes.

In this study, we aimed to identify and validate novel molecular targets for drug sensitivity analysis by revealing the immune profile of CRC through scRNA-seq data analysis. We explored the functional roles and differentiation trajectories of tumor cell subtypes, established a prognostic model based on genetic risk scores, and identified relevant prognostic genes. Furthermore, we validated *DLX2*, a gene strongly correlated with poor prognosis, through *in vitro* experiments. This comprehensive approach enhances our understanding of CRC progression and metastasis, providing actionable strategies to optimize immunotherapeutic approaches and improve patient outcomes.

## 2 Materials and methods

### 2.1 Data source

The CRC Single‐Cell RNA Sequencing (scRNA-seq) data were obtained from the GEO website (https://www.ncbi.nlm.nih.gov/geo/), GSE number was GSE161277. The dataset includes patient-matched blood, normal, para-cancer, polyp (adenoma), and cancer tissue. As we used publicly available data from databases, we did not need to assign an ethical approval number for this study.

### 2.2 scRNA-seq

Gene expression data was imported into the R software (version 4.2.0) and analyzed using the Seurat R package (version 4.3.0) (40). Low-quality cells were rejected: (1) 300 < nFeature < 7,500; (2) 500 < nCount < 100,000; (3) mitochondrial gene expression not exceeding 25% of the total number of genes in the cell; (4) erythrocyte gene expression not exceeding 5% of the total number of genes in the cell.

The harmony R package (v.0.1.1) (41, 42) was used to eliminate the batch effect between samples (43–45). The dimensionality reduction parameter (dim value) was set to 30 and the resolution parameter was set to 1.2. All gene expression data were normalized and scaled using the NormalizeData and ScaleData functions in the Seurat R package (v.3.1.4) (46). The “FindVariableFeautres” function (47) was used to select the top 2,000 most variable genes for PCA (48–51). Cells were divided into clusters based on the top 30 principal components (PCs) using FindClusters at a resolution of 1.0. The top 30 essential PCs were selected for uniform manifold approximation and projection (UMAP) downscaling and gene expression visualization (52, 53).

### 2.3 Cell type recognition

We conducted differential expression analysis of all genes in cell clusters using Seurat’s FindAllMarkers function to identify the marker genes in each cluster (54–56). An adjustment of P-value< 0.05, expression percentage > 0.25, and | log2 [fold change (FC)] | > 0.25 were taken as threshold standards for identifying marker genes. Afterwards, different cell clusters were identified and annotated by the singleR package based on the composition patterns of the marker genes and were then manually confirmed and corrected with the CellMarker database.

### 2.4 Cancer preferences analysis

To assess the predilection of TCs subtypes for cancer, odds ratios (OR) were calculated using the following computational methods (57).

### 2.5 Assessment of cell stemness

AUCell (58) is a method for identifying cells with active genes in scRNA-seq data. It takes a gene set as input and outputs the “activity” of that gene set in each cell. It was used in this paper to rate the stemness of subtypes of TCs. Cell stemness was assessed through the utilization of the CytoTRACE R package (version 0.3.3) (59), allowing the temporal order of cell differentiation to be inferred (60, 61).

### 2.6 Trajectory analysis of TCs subtypes

Single-cell pseudotime trajectories were constructed using the Monocle 2 algorithm (53, 62), an R package designed for single-cell trajectories by Qiu et al (63). This algorithm employs a machine learning technique to narrow down the high-dimensional expression spectrum to a low-dimensional space, visualized as a UMAP plot. Single cells were cast into this space and sorted into a trajectory with branching points. A dynamic expression heatmap was constructed using the plot_pseudotime_heatmap function. Furthermore, differential expression analysis between branches was performed using the plot_genes_branched_heatmap function.

The slingshot R package (version 2.6.0) was used to infer cell lineages and pseudotimes (64). It defined lineage structure using clustering-based minimum spanning trees (MSTs) and applications synchronized master curves to fit branching curves for these lineages. The getCurves function was used to obtain smooth trajectory curves.

### 2.7 Enrichment analysis of cellular subtypes

Using the Gene Ontology (GO) (52, 65–67), Genome Enrichment Analysis (GSEA) (http://software.broadinstitute.org/gsea/msigdb) and Kyoto Encyclopedia of Genes and Genomes (KEGG) (68) tools in the ClusterProfiler R package (version 4.6.0) (63), differentially expressed genes (DEGs) were enriched and analyzed. Significance of GO terms was determined based on an adjusted P < 0.05 (69, 70).

### 2.8 Gene regulatory network analysis

To investigate the top 5 transcription factors (TFs) with the most prominent expression changes in each TCs subtypes, single-cell regulatory network inference and clustering analyses were performed using the pySCENIC R package (version 0.10.0) in Python (version 3.7). Initially, GRNBoost was leveraged to identify potential target genes for each TFs. Then, DNA-motif analysis helped to determine potential direct binding targets. Lastly, the activity of regulon in the cells was scored by AUCell, and the top 5 TFs with the highest score were identified. The human gene-motif rankings were derived from https://resources.aertslab.org/cistarget/. Regulon modules were then identified according to the Connection Specificity Index (CSI) to confirm specific associating partners (71). Hierarchical clustering with Euclidean distance was then conducted to identify different regulon modules. We then constructed a regulator association network using 0.65 as a cutoff to investigate the relationship between different regulators.

### 2.9 Cell communication analysis

The CellChat R package (version 1.6.1) was utilised to infer sophisticated intercellular interactions and construct regulatory networks based on ligand-receptor levels (72). It utilizes the “netVisualDiffInteraction” function to indicate differences in the strength of intercellular communication and the “IdentifyCommunicationPatterns” function to indicate an estimate of the number of communication patterns. We used a significance threshold setting with a P-value cutoff of 0.05 to predict cell-cell interactions between different cell types.

### 2.10 Construction and validation of the prognostic model

To elucidate the impact of CRC-associated TCs on predicting patient survival, we employed marker genes of critical tumor cell subtypes as predictive factors for the construction of a prognostic model. Employing univariate Cox analysis and LASSO regression analysis (73–75), we identified the most significant prognostic genes. Multivariate Cox regression analysis was then conducted to calculate the risk coefficients for each prognostic gene (76–78), enabling the establishment of a risk score model:

Risk score = $\sum_{\text{i}}^{\text{n}}\text{Xi}\times \text{Yi}$ , where X represents the coefficient and Y represents the gene expression level.

Patients were categorized into low-risk and high-risk groups based on the optimal cutoff value calculated using the “surv_cutpoint” function. To observe the prognostic outcomes in different patient cohorts, we further utilized the R package “Survival” (version 3.3.1) for survival analysis of the constructed risk score model and visualized survival curves using the “ggsurvplot” function. The accuracy and calibration of the predictive model were evaluated and calibrated using the “timeROC” package (version 0.4.0) to plot ROC curves. The AUC values for the time-dependent ROC curve were computed at different time intervals to evaluate the model’s predictive accuracy over time (79).

CIBERSORT R package is a machine learning approach, which estimates the abundance of cell clusters in bulk RNA-seq data (80). We used this tool to digitally purify the transcriptomes of individual cell clusters from bulk data without isolating single cells. We extracted the transcripts per million (TPM) normalization datasets of selected cell types including *CD8*+ T cells, fibroblasts, myeloid cells and epithelial cells to create the signature matrix in 1000 permutations and without batch correction. We then divided the CRC patients available in the TCGA database into training and testing cohorts at a 1:1 ratio using survival-based randomisation and estimated the proportion of each cell cluster in the training and testing cohorts, respectively, using CIBERSORT. Notably, the bulk RNA-seq data from TCGA was initially normalized to TPM values.

### 2.11 Cell culture

NIC-H716 and SW837 cell lines were obtained from American Type Culture Collection (ATCC). NIC-H716 cell line was cultured in RPMI 1640 medium containing 10% fetal bovine serum (FBS) and 1% penicillin-streptomycin under standard conditions (37°C, 5% CO2, 95% humidity). SW837 cell line was cultured in RPMI 1640 medium containing 10% fetal bovine serum (FBS), 1% penicillin-streptomycin under standard conditions (37°C, 5% CO2, 95% humidity) (81).

### 2.12 Cell transfection

*DLX2* knockdown was achieved using small interfering RNA (siRNA) constructs from GenePharma (Suzhou, China). Transfection was performed following the manufacturer’s instructions of Lipofectamine 3000 RNAiMAX (Invitrogen, USA). Cells were seeded at 50% confluency in 6-well plates and transfected with negative control (si-NC) and knockdown constructs (Si-*DLX2*-1 and Si-*DLX2*-2). Lipofectamine 3000 RNAiMAX (Invitrogen, USA) was used for each transfection.

### 2.13 Cell viability assay

Cell viability of transfected NIC-H716 and SW837 cells was assessed using CCK-8 assay. Cells were seeded in 96-well plates at a density of 5×10³ cells per well and cultured for 24 hours. After adding 10μL of CCK-8 reagent (A311-01, Vazyme) per well, plates were incubated at 37°C in the dark for 2 hours. Absorbance at 450nm was measured using a microplate reader (A33978, Thermo) on days 1, 2, 3, and 4 post-transfection. Mean OD values were calculated and plotted (82).

### 2.14 Wound-healing assay

Stably transfected cells were seeded in 6-well plates and grown to confluence. A sterile 200μL pipette tip was used to scratch each well, which was then washed with PBS to remove cell debris and incubated in serum-free medium. Images of the scratches were captured at 0 hours and 48 hours, and the width of the scratch was measured using Image-J software (21).

### 2.15 Transwell assay

Cells were starved in serum-free medium for 24 hours prior to the experiment. Following treatment with Matrigel (BD Biosciences, USA), cell suspension was added to the upper chamber of Costar plates, with serum-containing medium in the lower chamber. Cells were incubated for 48 hours in a cell culture incubator. Following cultivation, cells were fixed with 4% paraformaldehyde and stained with crystal violet to assess their invasive capacity.

### 2.16 5-Ethynyl-2’-deoxyuridine proliferation assay

Transfected NIC-H716 and SW837 cells were seeded at 5×10³ cells per well in 6-well plates and cultured overnight. Subsequently, a 2x EdU working solution was prepared by mixing 10 mM EdU with serum-free medium. After a 2-hour incubation at 37°C, cells were washed with PBS, fixed with 4% paraformaldehyde for 30 minutes, permeabilized with 2 mg/mL glycine and 0.5% Triton X-100 for 15 minutes, and then stained with a mixture of 1X Apollo and 1X Hoechst 33342 for 30 minutes at room temperature. Cell proliferation was quantified using fluorescence microscopy.

## 3 Results

### 3.1 Single-cell mapping of CRC was obtained by scRNA-seq, yielding 8 cell types

**Figure 1:**
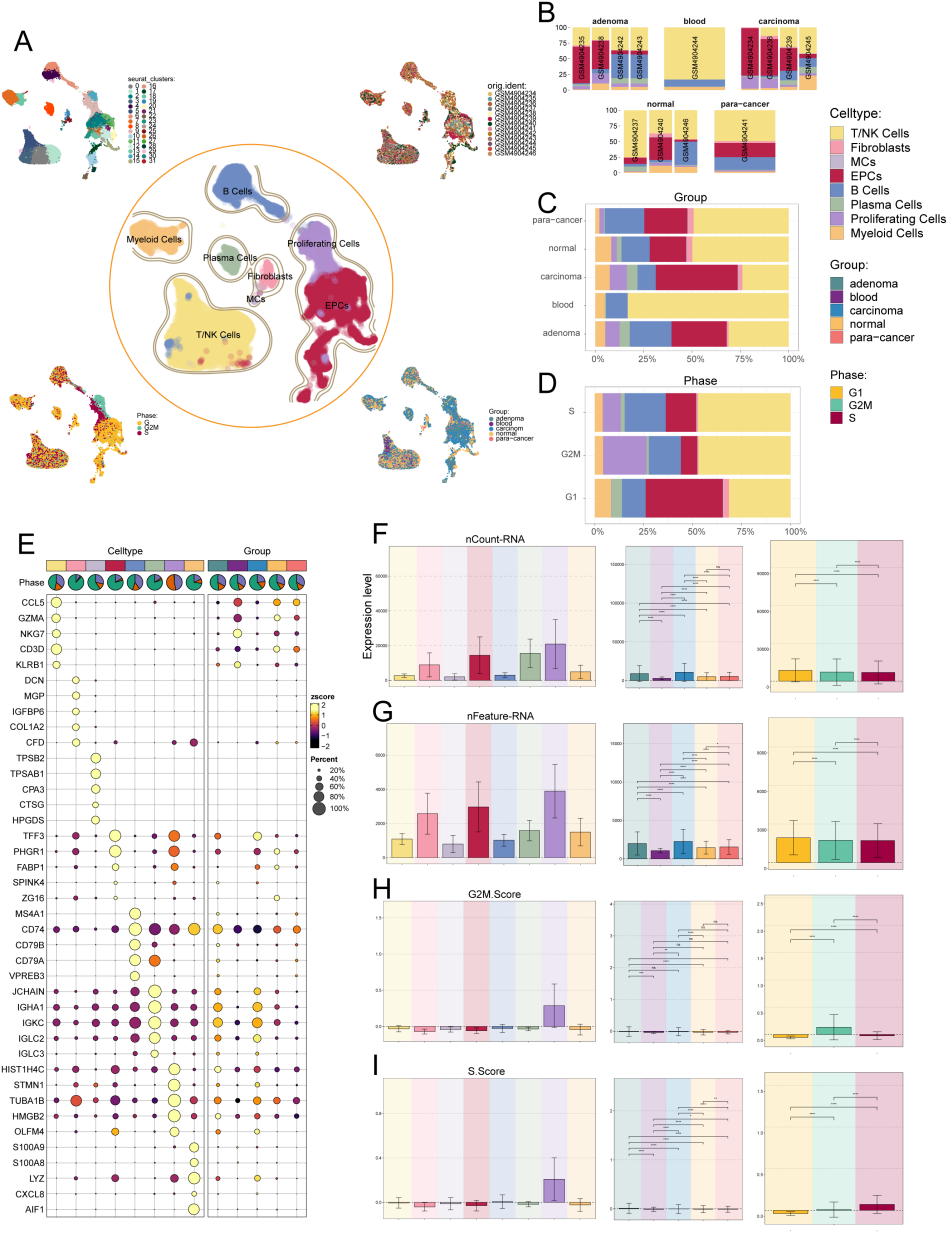
Single-cell analysis was carried out on CRC, including 32 cell populations, 8 cell types, and 5 tissue sources. **(A)** UMAP plot showed the 32 clusters of cells in CRC patients (top left); UMAP plot showed the 8 major cell types obtained by dimensionality reduction clustering of cells in CRC (middle); UMAP plot showed the distribution of sample sources (top right), cell phases (bottom left), and 5 tissue sources (bottom right) of all cells. Each point corresponded to a single cell colored according to cell cluster or cell type. **(B-D)** The bar graphs showed the proportion of different cell types in sample sources **(B)**, tissue sources **(C)** and cell phases **(D)** respectively. **(E)** Bubble plot showed differential expression of top 5 marker genes in CRC across 8 cell types. Bubble colors were based on normalized data (zscore), and sizes indicated the percentage of genes expressed in each cell type. **(F-I)** Bar graphs revealed the expression levels of nCount-RNA, nFeature-RNA, G2M.Score, and S.Score in different cell types, tissue sources and cell phases. ns stands for P>0.05 and the results are not statistically significant, * stands for P ≤ 0.05, ** stands for P ≤ 0.01, *** stands for P ≤ 0.001 and **** represents P ≤ 0.0001, a statistically significant difference.

To identify the major cell types during CRC progression, we collected 13 tissue samples containing adenoma, blood, carcinom, normal and para-cancer from 4 CRC patients on GEO for scRNA-seq. In addition, we checked the quality and completeness of the raw data. We then excluded genes in the samples that did not reach the minimum expression threshold. After initial quality control and removal of batch effects, we maintained a total of 42,133 cells (**Figure 1A**). We classified these 42,133 cells into 32 cell clusters by dimensionality reduction clustering. Based on cellular genetic profiles and typical markers, we finally identified the 32 cell clusters into 8 cell types, including T and NK cells (*CD3D*), fibroblasts (*DCN*), mast cells (MCs, *TPSB2*), epithelial cells (EPCs, *PHGR1*), B cells (*CD74*), plasma cells (*JCHAIN*), proliferating cells (*TUBA1B*) and myeloid cells (*LYZ*). From the UMAP plots and the bar plots we could learn that from normal to para-cancer to adenoma and carcinoma, the proportion of EPCs and proliferating cells roughly increases gradually, in contrast to a gradual decrease in the proportion of T and NK cells, and the proportion of B cells from normal to para-cancer to adenoma was gradually increasing but decreased in carcinoma; for cell cycle, most EPCs were in the G1 phase, which was the pre-DNA synthesis period with active material metabolism, aiming to prepare the material and energy for DNA replication in the next stage, the S phase (83), whereas T and NK cells were evenly distributed in all three periods, and proliferating cells were only present in S and G2M phases, synthesizing DNA as well as making sufficient preparations for mitosis, in line with their proliferative properties (**Figures 1B–D**). The bubble plot showed us the first five markers used to distinguish cell types, and the results were consistent with immunology (**Figure 1E**). The bar graphs showed the expression levels of nCount-RNA, nFeature-RNA, G2M.Score and S.Score for different cell types, tissue sources and cell cycle, respectively. From the results, it could be seen that proliferating cells had the highest expression levels of all four items, while EPCs, fibroblasts and plasma cells ranked behind them. In the tissue source, from normal to para-cancer to adenoma to carcinoma, the expression of both nCount-RNA and nFeature-RNA gradually increased, and there were statistically significant differences between all groups (**Figures 1F–I**).

Among the above cell types, EPCs attracted our attention. EPCs not only have relatively abundant RNA counts, but also tend to aggregate gradually during the developmental stages of cancer, whereas the tumor cells of CRC originate from the intestinal epithelial cells of the colon and rectum (84). The tumor cells are the most fundamental cause of tumors, so we next turned our attention to the study of CRC’s tumor cells.

### 3.2 Single-cell sequencing of TCs in the CRC

**Figure 2:**
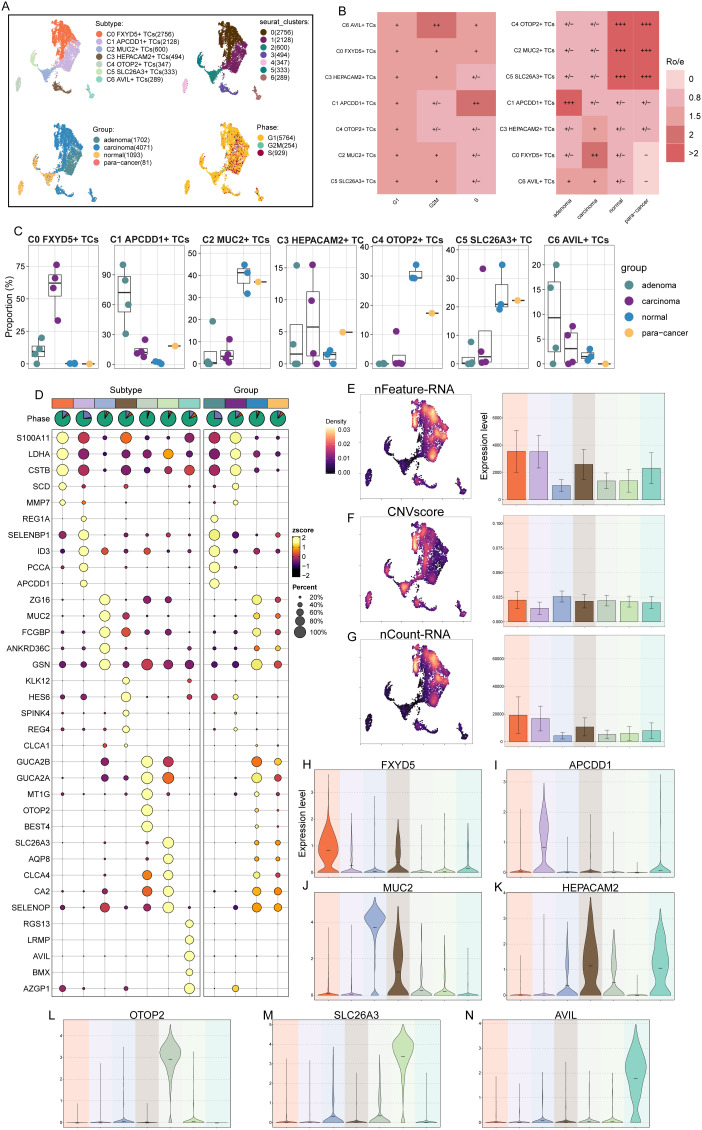
7 TCs subtypes were identified by different markers. **(A)** UMAP plot demonstrated the 7 cell subtypes of TCs in CRC patients and the number of cells in each cell subtype (top left); UMAP plot demonstrated the distribution of 7 clusters (top right), 4 tissue sources (bottom left) and cell phases (bottom right) in the 7 TCs subtypes respectively. Each point corresponded to a single cell colored according to cell different groups. **(B)** Cell phases (left) and tissue sources (right) preference of each TCs subtype estimated by Ro/e score. **(C)** The box line plots showed the proportion of different tissue sources in each TCs subtype. **(D)** Bubble plot showed differential expression of top 5 maker genes in 7 TCs subtypes. Bubble colors were based on normalized data (zscore) and sizes indicated the percentage of genes expressed in each subtype. **(E-G)** UMAP and bar plots revealed the expression levels of nFeature-RNA, CNVscore and nCount-RNA in different TCs subtypes. **(H-N)** Violin plots showed the expression levels of named gene in each of the TCs subtypes, in order of subtype nomenclature.

Next, we identified malignant tumor cells from epithelial cells using inferCNV (**Supplementary Figure S1**). Due to the absence of blood vessels in the epithelial tissues, we removed the blood tissues and compiled scRNA-seq data from the remaining four tissues in the CRC for further subclustering analysis against tumor cells. The analysis yielded seven clusters, resulting in seven different tumor cell subtypes: C0 *FXYD5*+ TCs, C1 *APCDD1*+ TCs, C2 *MUC2*+ TCs, C3 *HEPACAM2*+ TCs, C4 *OTOP2*+ TCs, C5 *SLC26A3*+ TCs, and C6 *AVIL*+ TCs, and showed the distribution of the various tissue origins and cellular period distribution in the subtypes (**Figure 2A**). Ro/e preference plots combined with box line plots showed that C6 *AVIL*+ TCs preferred the G2M phase, while C1 *APCDD1*+ TCs preferred the S phase, and were favored by adenoma tissues, whereas in contrast, C2 *MUC2*+ TCs, C4 *OTOP2*+ TCs, and C5 *SLC26A3*+ TCs were found overwhelmingly in the normal tissue and para-cancer tissues, whereas C0 *FXYD5*+ TCs preferred carcinoma tissues (**Figures 2B, C**). The above tissue distribution features suggested that C1 *APCDD1*+ TCs may be a watershed for colorectal progression from adenoma to malignancy, whereas C0 *FXYD5*+ TCs was a subtype that had become cancerous. The top five marker genes obtained by enrichment in different TCs subtypes were next visualized by bubble plots. We could see that *S100A11* was significantly enriched in C0 *FXYD5*+ TCs, whereas overexpression of *S100A11* in cytoplasmic and nuclear subcellular compartments was associated with tumor metastasis and poor prognosis in patients with CRC and meta-analyses demonstrated that the expression level of *S100A11* in CRC tissues significantly elevated in CRC tissues, a marker gene for clinical use in identifying CRC (85, 86). Meanwhile, *SELENBP1* was also noteworthy, as it was highly expressed in C1 *APCDD1+* TCs, and studies had shown that it was down-regulated in CRC but not in normal tissue or adenoma tissue (87), a typical oncogene that inhibits colorectal cancer progression by suppressing the EMT (88), whereas it was low-expressed in C0 *FXYD5*+ TCs, reflecting its intermediary role in the progression from C1 *APCDD1*+ TCs to C0 *FXYD5*+ TCs (**Figure 2D**). From the UMAP plots and bar graphs, we could tell that the C0 *FXYD5*+ TCs and C1 *APCDD1*+ TCs subtypes had an abundant number of RNA, as well as a higher CNVscore for C0 *FXYD5*+ TCs and C2 *MUC2*+ TCs (**Figures 2E–G**).

Finally, the violin plots showed the marker genes of each TCs subtype in turn (**Figures 2H–N**), which had shown corresponding expression advantages in their respective subtypes. *FXYD5* in C0 *FXYD5*+ TCs deserved our attention, as it had been shown to activate TGF-β/SMAD signaling and drive EMT to promote ovarian cancer progression, and to form a positive feedback loop to drive EMT during the progression of ovarian cancer and to promote tumor growth and metastasis (89); meanwhile, recent study also showed that *FXYD5* was upregulated in various tumor types and positively correlates with tumor progression (90).

### 3.3 Analysis of stemness genes in TCs subtypes

**Figure 3:**
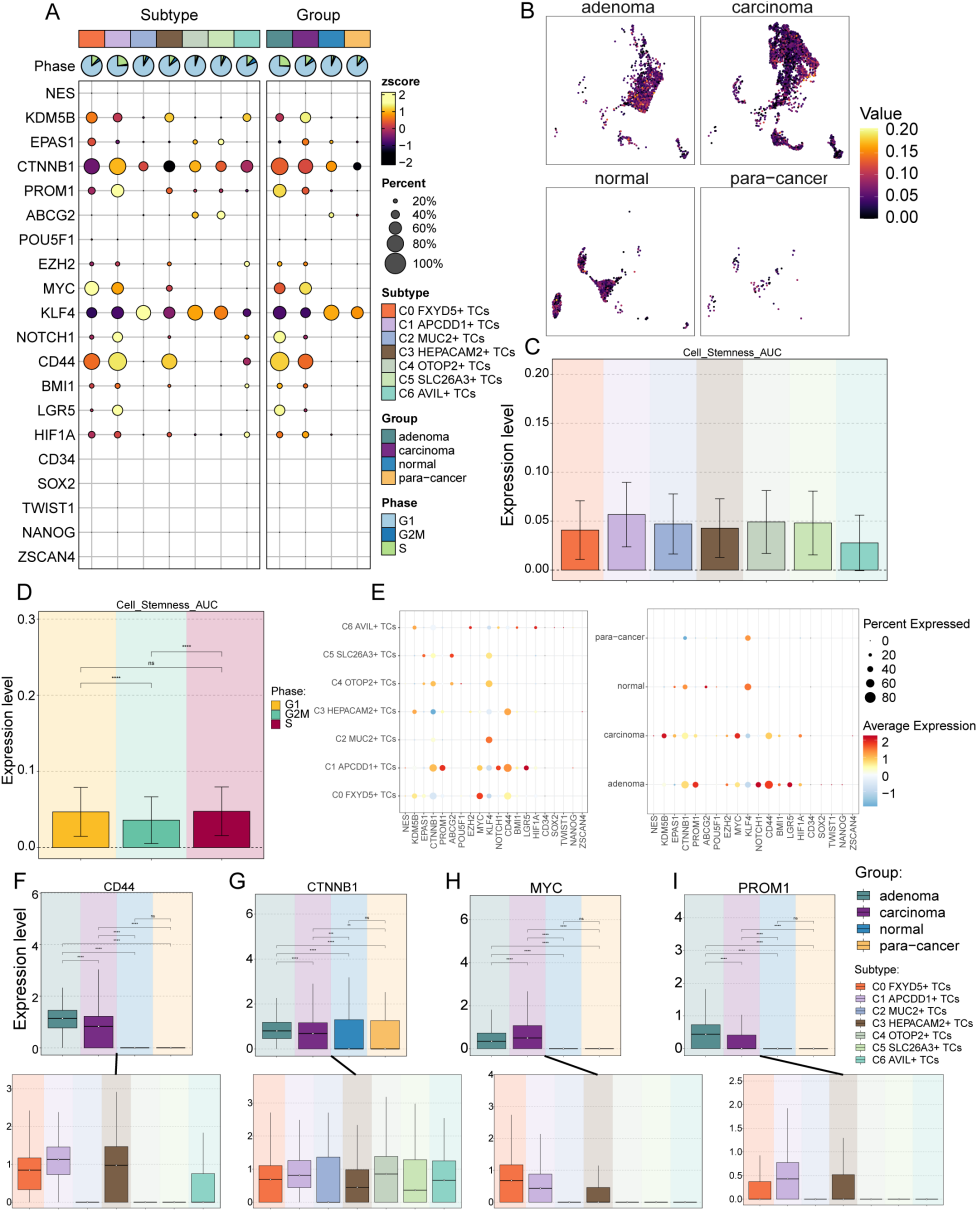
Analysis of cell stemness in TCs subtypes. **(A)** Bubble plot showed expression of stemness genes in 7 TCs subtypes. Bubble colors were based on normalized data (zscore) and sizes indicated the percentage of genes expressed in each subtype. **(B)** The UMAP plots showed the cell stemness AUC values for different tissue sources in TCs, respectively. **(C, D)** Bar graphs revealed the AUC values of TCs cell stemness in different subtypes **(C)** and cell phases **(D)** ns stands for P>0.05 and the results are not statistically significant and **** represents P ≤ 0.0001, a statistically significant difference. **(E)** Bubble plots demonstrated the expression of stemness genes in different cell subtypes (left) and tissue sources (right), where the colors indicated high or low average expression levels and sizes indicated the percentage of genes expressed in each subtype and tissue source. **(F-I)** Box line plots visualized the 4 stemness genes expressed in TCs tissue sources and subtypes. ns stands for P>0.05 and the results are not statistically significant, ** stands for P ≤ 0.01, *** stands for P ≤ 0.001 and **** represents P ≤ 0.0001.

To explore the expression of stemness genes in TCs subtypes and to comprehend their proliferative differentiation potential, we used bubble plot to illustrate the different expression of stemness genes in TCs subtypes (**Figure 3A**). The differential expression analysis was intended to provide a holistic view of stemness-related gene expression, including both high and low-expression markers, to fully capture the diversity of stemness-associated features across tumor cell subtypes. Results showed the corresponding expression of stemness genes *KDM5B, EPAS1, CTNNB1, MYC*, *KLF4* and *CD44* in TCs subtypes and different tissue sources, with *MYC* highly expressed in C0 *FXYD5*+ TCs and *PROM*1 in C1 *APCDD1*+ TCs. Following this, UMAP plot showed the AUC scores of cells stemness in different tissue sources, which were significantly higher in adenoma and carcinoma than in normal and para-cancer (**Figure 3B**). Next, bar graphs showed the AUC scores of stemness genes in different TCs subtypes and in the cell cycle, respectively (**Figures 3C, D**). We next enumerated the stemness genes enriched in different TCs subtypes and different tissue sources using bubble plots, respectively, and the results remained that *MYC* was significantly enriched in C0 *FXYD5*+ TCs, and for the tissues, *PROM1*, *NOTCH1*, and *CD44* were enriched in adenoma, whereas *KDM5B* and *MYC* were enriched in carcinoma. Stemness genes were also enriched in normal and para-cancer, but at lower levels, which suggested that adenoma and carcinoma tissues had a higher proliferative potential (**Figure 3E**). Finally, we visualized and compared the stemness genes with higher expression in C0 *FXYD5*+ TCs and C1 *APCDD1*+ TCs using box line plots, and the results further validated the previous results, showing a stronger proliferative capacity in C0 *FXYD5*+ TCs and C1 *APCDD1*+ TCs as well as adenoma and carcinoma (**Figures 3F–I**).

### 3.4 Construction of single-cell trajectories of TCs using Monocle and Slingshot for pseudotime analysis

**Figure 4:**
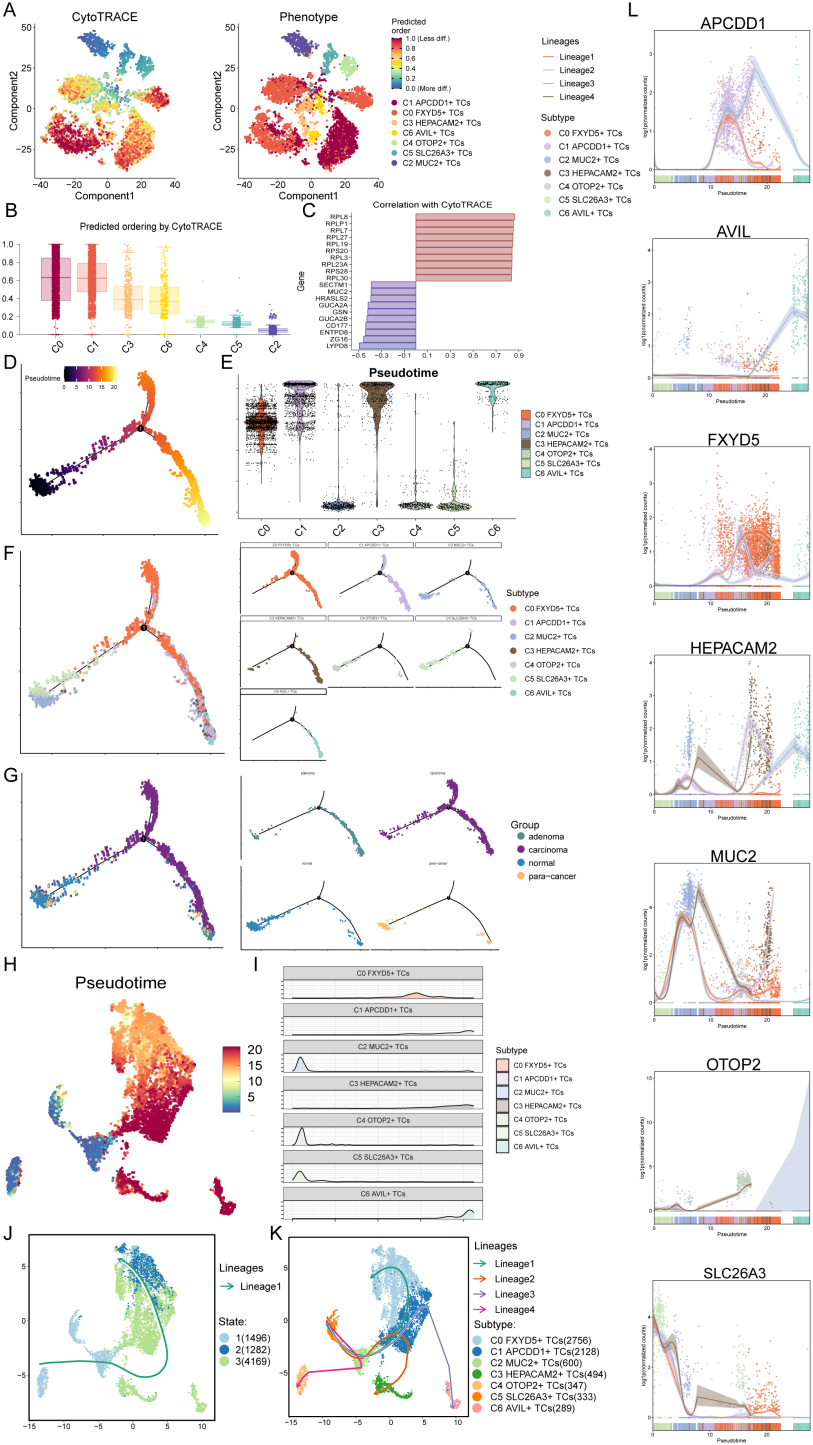
CytoTRACE and SlingShot analyses demonstrated the developmental trajectories of different subtypes of TCs. **(A)** The left panel demonstrated the distribution of TCs CytoTRACE scores. The color represented high or low cell stemness. The right panel indicated the distribution of TCs subtypes. The color represented different TCs subtypes. **(B)** Box line plot ranked the stemness of TCs subtypes according to CytoTRACE. **(C)** The bar graph showed the correlation of different genes with CytoTRACE, where higher than 0 indicates a positive correlation and less than 0 indicates a negative correlation. **(D)** Pseudotemporal trajectory plot demonstrated monocle-predicted differentiation trajectories of TCs. The distribution of pseudotime order by Monocle were shown. **(E)** Violin plots demonstrated the pseudotime distribution and density of different subtypes of TCs. **(F, G)** The pseudotime distribution of subtypes **(F)** and tissue sources **(G)** of TCs by Monocle were shown respectively. **(H)** Demonstration of the distribution of slingshot-predicted TCs differentiation trajectories among all TCs by UMAP plot. Plotting each spectrum according to the pseudotime value to infer the result, the color from blue to red indicates the pseudotime from naïve to mature. **(I)** Ridgeline plots demonstrated the pseudotime distribution and density of different subtypes of TCs. **(J)** The distribution of differentiation trajectory of 3 states fitted by the pseudotime order in all tumor cells. **(K)** The distribution of four differentiation trajectories of 7 TCs subtypes fitted by the pseudotime order in all tumor cells. **(L)** Dynamic trend plots demonstrated the trajectories of named genes of 7 cell subtypes of tumor cells changing on four lineages obtained after slingshot visualization.

Based on the above results, in order to further clarify the differentiation status of TCs subtypes, we first used CytoTRACE to clustered all tumor cells, and obtained UMAP plots and box line plots of cell stemness prediction ordering (**Figures 4A, B**). From these three plots, we could learn that the C0 *FXYD5*+ TCs and C1 *APCDD1*+ TCs subtypes were in a high stemness and low differentiation state, which was consistent with the above results (**Figure 3**). Next, the bar graphs showed the genes that correlated with the CytoTRACE results, where greater than 0 was a positive correlation and vice versa was a negative correlation (**Figure 4C**). We then performed Monocle analysis of tumor cells to obtain a pseudotime atlas of tumor cell subtypes, colored by the order of pseudotime, different subtypes, and different tissue sources (**Figures 4D–G**). First, the pseudotime order speculated that the developmental trajectory started at the bottom left corner and branches out to two more trajectories when it reached state 1. Also in conjunction with the subtypes of TCs, C2 *MUC2*+ TCs, C4 *OTOP2*+ TCs, and C5 *SLC26A3*+ TCs subtypes were concentrated in early developmental trajectories, whereas C1 *APCDD1*+ TCs, C3 *HEPACAM2*+ TCs, and C6 *AVIL*+ TCs were concentrated in late developmental trajectories, and unlike C0 *FXYD5*+ TCs subtype, it was found in mid- to late-developmental trajectories; for the tissue origin, normal and para-cancer tissues were present at the initial developmental stage, which later i.e., developed into the adenoma and carcinoma.

Furthermore, we then performed Slingshot analysis on tumor cells, which resulted in a pseudotime speculative map colored by pseudotime order and based on the UMAP plot obtained earlier (**Figure 4H**). Combined with the ridge plot, the analysis showed that, again, C2 *MUC2*+ TCs, C4 *OTOP2*+ TCs, and C5 *SLC26A3*+ TCs subtypes were at the anterior end of the developmental trajectory, while the rest of the subtypes were located in the middle to posterior (**Figure 4I**). By observing how Slingshot’s spectral differentiation trajectories were plotted, we could learn that tumor cells go from state 1 to state 3 to state 2; at the same time, this result yielded four spectral trajectories based on cell subtypes, as follows: Lineage1: C5 to C2 to C1 to C0; Lineage2: C5 to C2 to C1 to C3; Lineage3: C5 to C2 to C1 to C6; Lineage4: C5 to C2 to C4 (**Figures 4J, K**). The differences between the four trajectories were mainly in the late stage, with lineage1 ending at C0, lineage2 at C3, lineage3 at C6, and lineage4 at C4, where C0 not only had a preference for, but also a high percentage of tumor tissue. Hence, we predicted that lineage1 represented the lineage of differentiation of TCs associated with CRC tumorigenesis. Furthermore, we noted that lineage1 passed through C1, which was predominantly organized as adenoma, a precancerous, benign tumor, and previous studies had reported that participants with positive diagnostic colonoscopy results and advanced adenomas had a significantly increased risk of colorectal cancer compared to participants without adenomas (91). Therefore, studying how C1 progresses to C0 and the key factors that drive the epithelium to change from benign to malignant has important implications for how to stop CRC from occurring and worsening. Finally, the dynamic trends plots displayed the expression changes and distribution of marker genes for TCs subtypes across the four differentiation trajectories in pseudotime (**Figure 4L**).

### 3.5 All TCs subtypes were analyzed for enrichment, especially C0 *FXYD5*+ TCs

**Figure 5:**
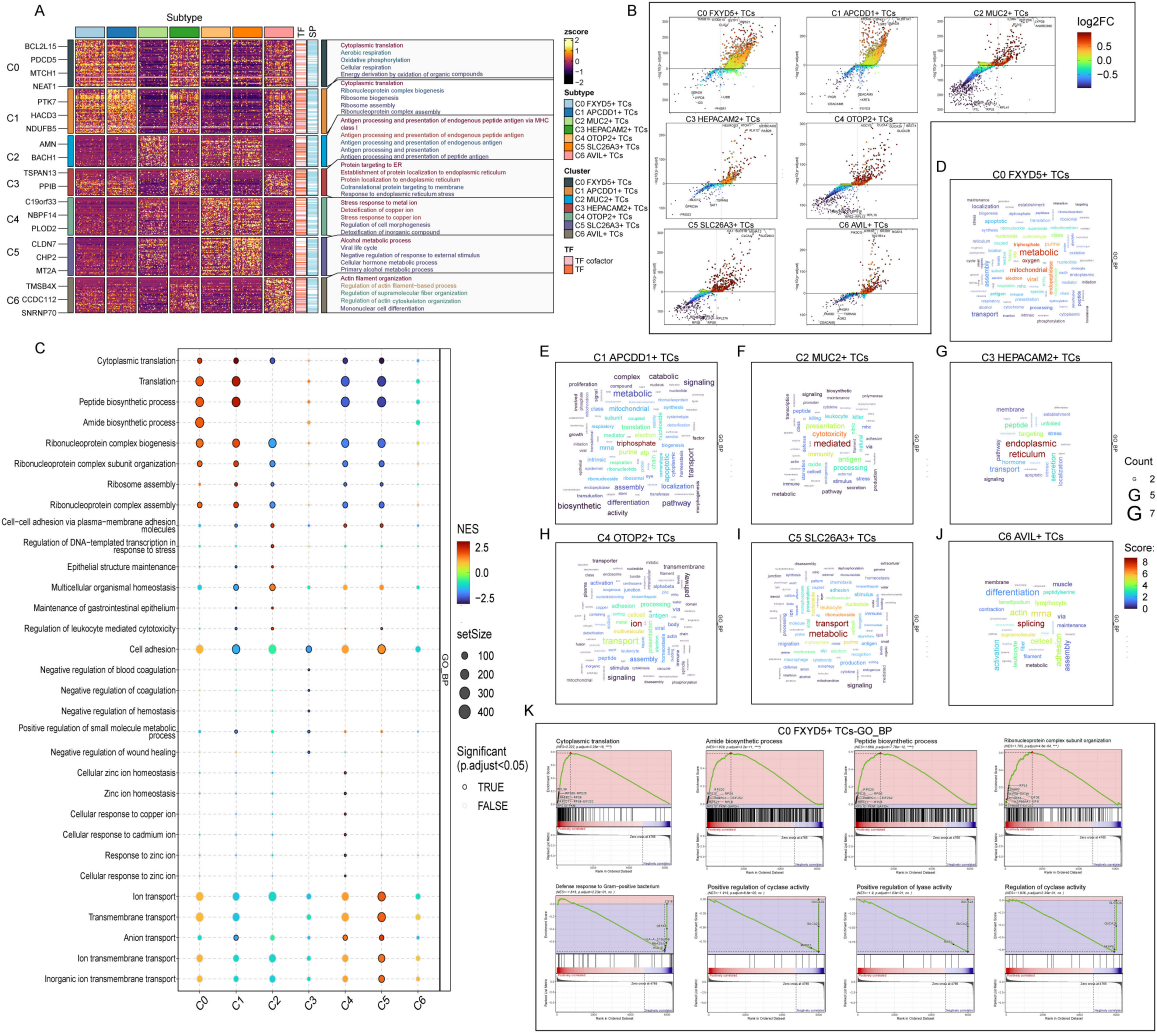
Functional enrichment analysis results of differentially expressed genes in 7 TCs subtypes. **(A)** The heatmap showed the GO-BP enrichment term scores. zscore > 0 was positive enrichment and < 0 was negative enrichment. **(B)** Volcano plots showed differentially expressed genes in 7 subtypes. **(C)** GSEA analysis diagram of different pathways in each TCs subtype. NES > 0 was positive enrichment and < 0 was negative enrichment. NES, N stands for standardization, and ES for enrichment scores. **(D-J)** Word cloud diagrams demonstrated the activity of different pathways in TCs subtypes. **(K)** GSEA results among C0 *FXYD5*+ TCs.

Then, we performed GO-BP DEGs in TCs subtypes to reveal their enrichment in biological processes. Heatmap displayed the results of the top five enriched items in the seven TCs subtypes (**Figure 5A**). The C0 subtype was mainly associated with pathways such as cytoplasmic translation, aerobic respiration, oxidative phosphorylation, cellular respiration and energy derivation by oxidation of organic compounds; The C1 subtype was enriched in pathways such as cytoplasmic translation, ribonucleoprotein complex biogenesis, ribosome biogenesis, ribosome assembly and ribonucleoprotein complex; The C2 subtype revealed their close association with antigen processing and presentation of endogenous antigen, endogenous peptide antigen or peptide antigen; The C3 subtype showed enrichment in pathways such as protein targeting to endoplasmic reticulum (ER), establishment of protein localization to endoplasmic reticulum, cotranslational protein targeting to membrane and response to endoplasmic reticulum stress; The C4 subtype was enriched in pathways related to stress response to metal and copper ion, detoxification of copper ion and inorganic compound and regulation of cell morphogenesis; The C5 subtype mainly exhibited enrichment in pathways such as alcohol metabolic process, viral life cycle, negative regulation of response to external stimulus, cellular hormone metabolic process and primary alcohol metabolic process; The C6 subtype revealed pathways related to actin filament organization, regulation of actin filament-based process, supramolecular fiber organization and actin cytoskeleton organization and mononuclear cell differentiation. The enrichment items derived from the C0 subtype caught our attention, and the last four items were all related to cellular energy metabolism, which corresponds to the increased energy metabolism that occurred after cancerous transformation, e.g., in order to maintain proliferative and metastatic capacity, tumor cells increased their energy-producing pathways (92), and which we conjecture mediate cytoplasmic and mitochondrial translational homeostasis in order to modulate the respiratory capacity of the mitochondria to provide the tumor cells to provide energy to adapt to different environmental stresses and growth demands (93). This implied that C0 subtype was strongly correlated with tumor tissue development and progression. Next, we utilized volcano plots to demonstrate the spectrum of DEGs among TCs subtypes (**Figure 5B**). The results showed that the main DEGs upregulated in C0 subtype were *TMSB10*, *S100A10, CLIC1, GSTP1* and *RPS21*. Among them, *TMSB10, S100A10, CLIC1* and *GSTP1* have been shown in previous studies to have an oncogenic mechanism in the colorectum as well as to contribute to the poor prognosis of colorectal cancer, and *TMSB10* could be used as a minimally invasive serum tumor marker for the detection of CRC, while *CLIC1* and *GSTP1* may be potential prognostic biomarkers for patients with CRC (94–97).

In addition, the bubble plot showed the results of GSEA (**Figure 5C**). The results showed that the C0 subtype was significantly expressed in the pathways of translation, peptide biosynthetic process, amide biosynthetic process and ribonucleoprotein complex biogenesis. All these pathways indicated that energy metabolism as well as ribose and protein synthesis were actively taking place in the C0 subtype, providing a material basis for the rapid proliferation of tumor cells. The word cloud map showed the pathways enriched in DEGs in the TCs subtypes (**Figures 5D–J**). Consistent with the above results, it indicated that the C0 subtype was undergoing enriched metabolic and oxidative reactions and substance synthesis. Finally, we performed a GSEA of DEGs in the C0 subtype according to GO-BP (**Figure 5K**). We observed that pathways associated with cytoplasmic translation, amide biosynthetic process, peptide biosynthetic process and ribonucleoprotein complex subunit organization, which were consistent with the previous enrichment items, were upregulated in the C0 subtype. While pathways associated with defense response to gram-positive bacterium, positive regulation of cyclase activity and lyase activity and regulation of cyclase activity were down-regulated in the C0 subtype. Where for positive regulation of lyase activity decreases, it may lead to increased levels of degradative enzymes, which in turn are able to degrade proteoglycan basement membrane components, promoting increased tumor cell detachment from the primary tumor and local invasion, resulting in a poor prognosis (98). Thus, the items enriched by various methods in the C0 subtype were suggestive that the C0 subtype might be transforming into tumors with a more aggressive and poorer prognosis, and that we could incorporate this feature into risk group of the tumor population.

### 3.6 TFs regulate the oncogenesis of C0 *FXYD5*+ TCs subtype

**Figure 6:**
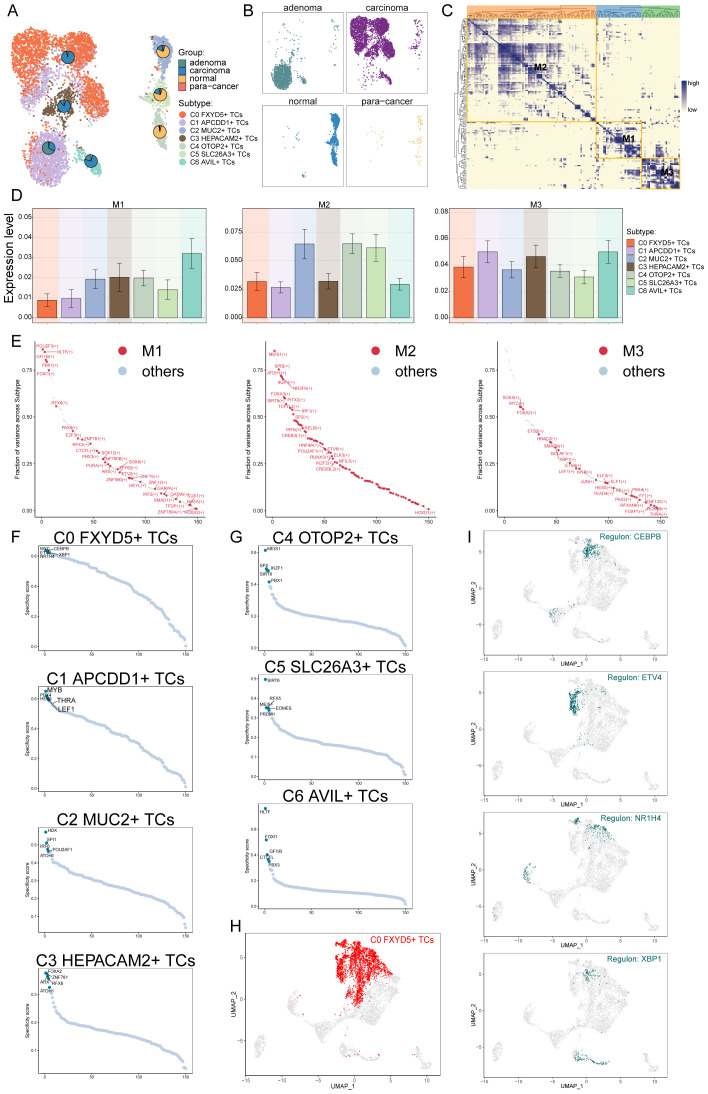
Identification of tumor cells Gene Regulatory Network. **(A, B)** UMAP plot visualization of all TCs based on regulator activity. Colored according to cell subtype. Pie charts showed the proportion of different tissue sources in TCs subtypes. **(C)** Based on the CSI matrix, three regulatory modules of TCs subtypes were identified. **(D)** Bar graphs illustrated the expression levels of different regulatory modules in subtypes of TCs. **(E)** In subtypes, the ranking of regulators in different regulation modules based on fraction of variance. **(F, G)** Rank for regulons in different TCs subtypes based on regulon specificity score (RSS). **(H)** C0 FXYD5+ TCs were highlighted in the UMAP plot (red). **(I)** Expression of key regulators *CEBPB, ETV4, NR1H4* and *XBP1* of C0 *FXYD5*+ TCs in all TCs.

TFs act directly on the genome to regulate gene transcription by binding to specific nucleotide sequences upstream of genes, thereby affecting the biological functions of cells. Here, we analyzed the gene regulatory network of the C0 subtype using scenic analysis. First, we performed a dimensionality reduction clustering of TCs based on the activities of regulatory factors and listed the results from different tissue sources (**Figures 6A, B**). Among them, the C0 subtype still mainly consisted of carcinoma tissues. Next, we observed the correlation between the TCs subtypes. Considering that different TFs can co-regulate the expression of certain genes, based on the connectivity specificity index (CSI), we categorized the TCs subtypes into three modules of regulatory factors: M1, M2 and M3 (**Figure 6C**). These three regulatory submodules showed TFs that may be cooperative with each other in gene regulation. As shown in the bar graphs, the M3 regulatory submodule may take a major regulatory role in the biological functions of the C0, C1, and C3 subtypes (**Figure 6D**). Meanwhile, the M3 regulatory submodule was also the dominant regulatory module for the C0 subtype (**Figure 6E**). By further analyzing the key regulators of different TCs subtypes, it was found that three of the five major regulators of the C0 subtype, *ETV4, MYC* and *XBP1*, were present in the M3 regulatory submodule (**Figures 6F, G**). The expression of TFs in the C0 subtype was labeled in the UMAP plot (**Figures 6H, I**). It is worth noting that previous studies have indicated that *CEBPB* regulates the bile acid receptor *FXR* to accelerate colon cancer progression by modulating aerobic glycolysis (99), corresponding to the analysis of its enrichment pathway; whereas, the expression of *ETV4* was significantly correlated with the depth of infiltration, lymphovascular and venous infiltration, lymph node and distant metastasis, and the stage of lymph node metastasis of the pathologic tumors in terms of progression and recurrence, which suggests an unfavorable prognosis (100); while the expression of *XBP1* could also promote the proliferation of colon cancer cells (101). These were all factors of poor prognosis of CRC.

### 3.7 CellChat analyzes the communication of all cells

**Figure 7:**
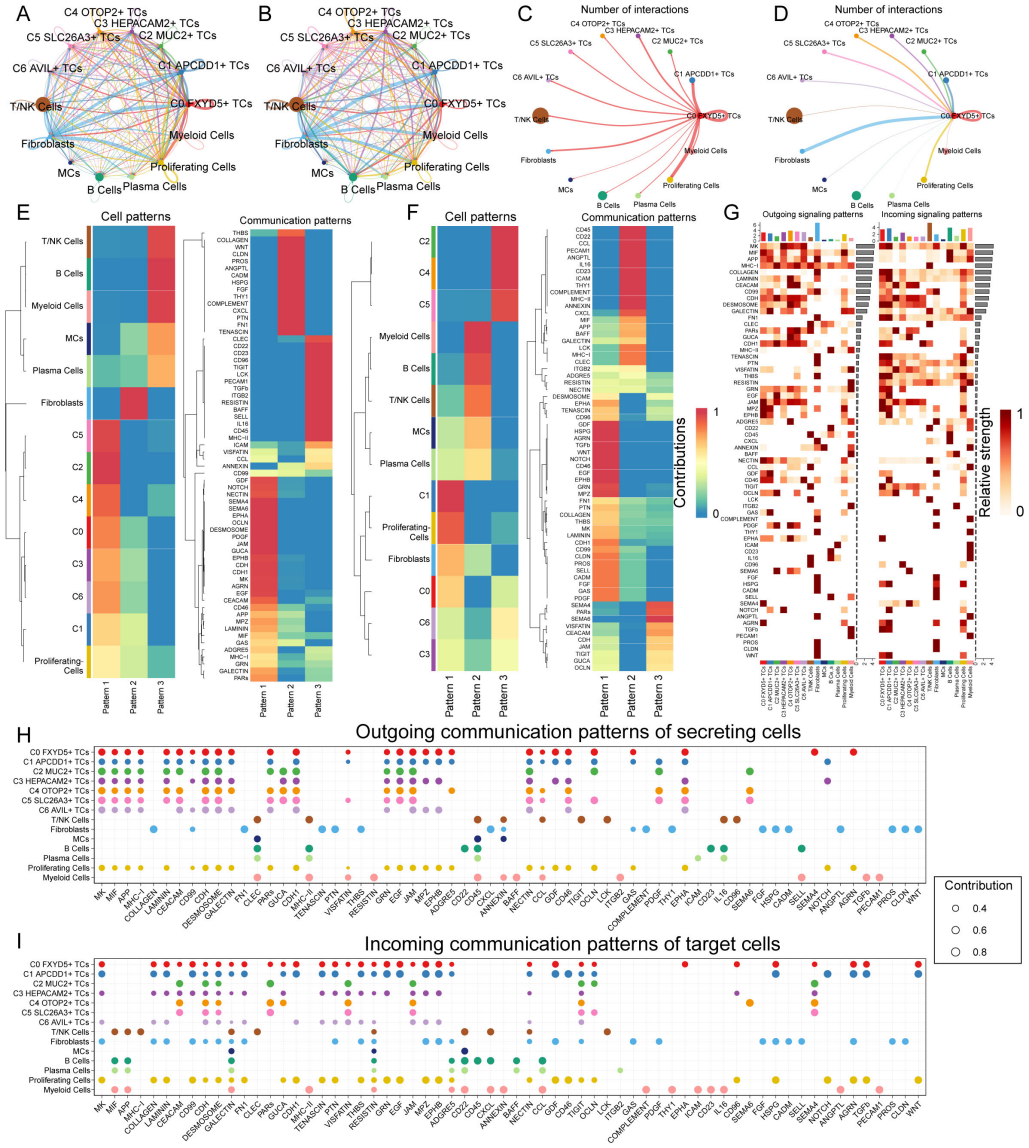
Communication analysis of all cells in CRC. **(A, B)** Circle plots showed the number **(A)** and strength **(B)** of interactions between all cells. **(C, D)** Circle plots of the number of cellular interactions with C0 *FXYD5*+ TCs as source **(C)** and target **(D)**. **(E, F)** Heatmap showed pattern recognition of outgoing cells **(E)**, and incoming cells **(F)** among all cells. **(G)** Heatmap showed ligands and receptors related to the incoming and outgoing signals of cell interactions. **(H, I)** Outgoing contribution bubble plot and incoming contribution bubble plot demonstrated the communication patterns between the secreting cells and target cells of CRC, the color of the dots indicated different cells and the size of the dots indicated the contribution of cells.

In order to specifically elucidate complex cellular responses, we intended to explore intercellular relationships and ligand-receptor communication networks for a better understanding of intercellular interactions. Through analyses via CellChat, we preliminarily established intercellular communication networks involving a variety of cells, such as proliferating cells, fibroblasts, T and NK cells, and different subtypes of TCs (**Figures 7A, B**). We also established a communication network with C0 *FXYD5*+ TCs as the source cells and target cells (**Figures 7C, D**). After building the intercellular communication network using CellChat analysis, we accounted for the number of interactions (expressed in terms of the thickness of the connecting lines between the two types of cells) and the strength of the interactions (expressed in terms of the weight of the connecting lines, with thicker lines indicating stronger interactions). This method helped to quantify the complexity and strength of the communication pathways between different cell types in the network. From the intricate communication network diagram, we could see that C0 *FXYD5*+ TCs sent positive communication signals to almost all cells, while fibroblasts and proliferating cells are the cell types that give it the most responses. Next, we used the gene expression pattern analysis method provided by CellChat to examine how cells and signaling pathways interact with each other (**Figures 7E, F**). First, we assessed the relationship between the inferred potential communication patterns and the population of cells secreting signaling molecules to decipher the outgoing communication patterns. Through our analysis, we identified three distinct signaling patterns: pattern 1 (tumor cells and proliferating cells), pattern 2 (fibroblasts), and pattern 3 (myeloid cells, B cells, plasma cells, MCs, and T and NK cells). Whereas for the incoming communication mode, the distinct signaling patterns: pattern 1 (C1 *APCDD1*+ TCs, proliferating cells, fibroblasts, C0 *FXYD5*+ TCs, C6 *AVIL*+ TCs and C3 *HEPACAM2*+ TCs), pattern 2 (myeloid cells, B cells, T cells and NK cells, MCs and plasma cells), and pattern 3 (C2 *MUC2*+ TCs, C4 *OTOP2*+ TCs and C5 *SLC26A3*+ TCs). Next, the heatmaps showed the signaling molecules of various types of cells under outgoing signaling patterns and under incoming signaling patterns, respectively (**Figure 7G**). Finally, to identify the major afferent and efferent signals associated with the seven TCs subtypes, we quantified the ligand-receptor network using CellChat (**Figures 7H, I**). With this approach, we could predict the major outgoing signals from secretory cells (signal senders) that release various cytokines or ligands. In addition, we evaluated on which cell types act as target cells (signal receivers) and how ligand-receptor-mediated communication between different cell types contributes to the progression of CRC. This analysis helps to illustrate how receptors on these cells are targeted by ligands released by the same or other cell types. In summary, we visualized signaling molecules between different cell types using multiple communication analyses that provide support for describing cellular communication.

### 3.8 Construction of risk score profiles and immune infiltration analysis

**Figure 8:**
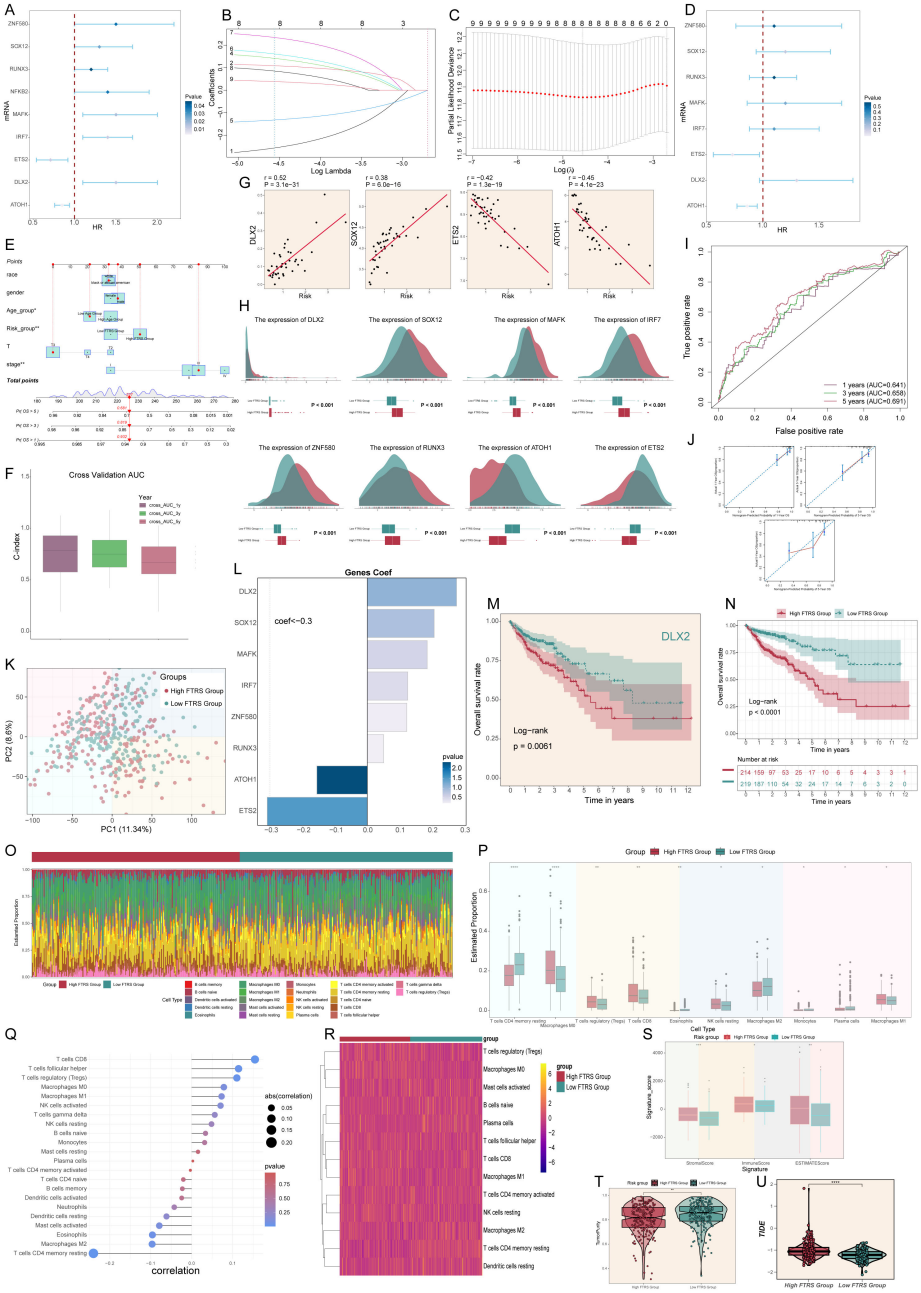
Constructed risk score profiles and immune infiltration and survival prognostic analyses for CRC. **(A)** Forest plot from univariate Cox regression analysis showcased statistically significant genes (P<0.05) with HR<1 indicating protective factors and HR>1 indicating risk factors. **(B, C)** Selection of eight prognostic-related genes (non-zero regression coefficients) was made via LASSO regression analysis, LASSO coefficient curve determined by optimal lambda **(B)**, with optimal parameter (lambda) determined through cross-validation **(C)**. **(D)** Forest plot of eight prognosis-related genes. **(E)** Column line graphs were used to predict patients’ prognosis at 1 year, 3 years and 5 years. For categorical variables, the importance of each variable was ranked according to the standard deviation of the column-line graph scale. To use a column line plot, individual patient-specific points are located on each variable axis. Red lines and dots are plotted upward to identify the point at which each variable is received; the sum of these points (280) is located on the Total Points axis, and a line is plotted downward to the Survival axis to identify the probability of overall survival. **(F)** Box line plots depict the C-index of the AUC values of the risk scores for predicting 1-, 3-, and 5-year survival. **(G)** Four genes that showed a significant correlation with the risk scores (*DLX2, SOX12, ETS2* and *ATOH1*). **(H)** The ridge and box line plots showed that gene expression of these four genes (*DLX2, SOX12, MAFK, IRF7*) was higher in the high-risk group compared to the low-risk group, while gene expression of these four genes (*ZNF580, RUNX3, ATOH1, ETS2*) was lower in the high-risk group. **(I)** ROC curves depict the sensitivity and specificity of the risk scores for predicting 1-, 3-, and 5-year survival. **(J)** Calibration curves for column charts predicting 1-, 3-, and 5-year overall survival. The OS predicted by the line plot model is plotted on the x-axis and the actual OS is plotted on the y-axis. **(K)** The PCA plot demonstrated the difference in the distribution of prognosis-related genes in the high FTRS and low FTRS groups. **(L)** Bar chart showed the coefficient (Coef) values of genes utilized for model construction. **(M)** OS curve of *DLX2*, a highly expressed gene screened by LASSO. **(N)** OS curves for different scoring subgroups in a cohort (high FTRS group and low FTRS group). **(O)** Proportion of each infiltrating immune cell type in the high- and low-FTRS groups were shown using CIBERSOFT. **(P)** Statistically different infiltrating immune cell type in the high- and low-FTRS groups were demonstrated using CIBERSOFT. * stands for P ≤ 0.05, ** stands for P ≤ 0.01 and **** represents P ≤ 0.0001. **(Q)** Lollipop chart of immune cell versus risk score. **(R)** Heatmap demonstrated the difference in expression of different immune cells in the two risk groups. **(S)** Stromal score, immune score, and estimate score were calculated for the high- and low-FTRS groups, respectively, using ESTIMATE. * stands for P ≤ 0.05, ** stands for P ≤ 0.01 and *** represents P ≤ 0.001. **(T)** TumorPurity was calculated using ESTIMATE for the high- and low-FTRS groups, respectively. ** stands for P ≤ 0.01. **(U)** Violin plot demonstrating the difference in Tumor Immune Dysfunction and Exclusion (TIDE) scores in the two risk groups. **** represents P ≤ 0.0001, a statistically significant difference.

Since we focused on the C0 *FXYD5*+ TCs subtype, we identified 9 genes that could be used as prognostic features based on the top 100 marker genes of the *FXYD5*+ TCs subtype by univariate Cox regression analysis (**Figure 8A**), of which *ETS2* and *ATOH1* were protective factors (HR < 1), and the other genes were risk factors. To address the problem of multicollinearity among these genes, we further screened them using LASSO regression analysis and multivariate regression analysis and finally identified 8 genes associated with prognosis (**Figures 8B–D**). The one that caught our attention was *DLX2*, which was the most dangerous prognostic factor among them with the largest HR. To further investigate the impact of *FXYD5* highly expressed TCs on CRC patients, we categorized patients in the TCGA cohort into high and low FTRS (*FXYD5*+ TCs risk score) groups based on the identified 8 prognostic genes. Subsequently, we created a Nomogram survival prediction model for OS in CRC patients using autonomous prognostic factors to predict the prognosis of CRC patients (**Figure 8E**). The model was validated and performed well in predicting the OS C index (**Figure 8F**). Subsequent prognostic gene versus risk correlation plots, as well as ridge and box line plots, illustrated the clear correlation of prognostic genes with risk and variability across risk groups (**Figures 8G–H**). It could be seen that the difference between the two groups for our gene of interest, *DLX2*, was statistically significant and was expressed higher in the high FTRS group than in the low FTRS group. Next, to assess the accuracy of risk scores in predicting 1-, 3- and 5-year survival in patients with CRC, we performed ROC curve analyses on the three cohorts. The results showed high predictive accuracy with AUC (1-year) = 0.641, AUC (3-years) = 0.658, and AUC (5-years) = 0.691 (**Figure 8I**). Moreover, calibration curves were plotted to show the consistency between predicted and observed values for 1-, 3- and 5-year OS in both the training and validation cohorts (**Figure 8J**), which showed good agreement. The PCA plot also provided further evidence of the discrete and differential character of the high and low FTRS groups (**Figure 8K**). With definitive evidence, we next analyzed the calculation of coefficient values for these prognostic genes (**Figure 8L**). Using Kaplan-Meier survival curves, we first confirmed the poor prognosis corresponding to high expression of *DLX2*, and then we further concluded that the survival outcome was worse in the high FTRS group (**Figures 8M, N**). *DLX2* was demonstrated to be associated with EMT in osteosarcoma in previous studies and its increased expression was associated with advanced gastric adenocarcinoma, but no article defining the relationship between *DLX2* and poor prognosis in colorectal cancer could be found yet (102, 103).

The CIBERSORT analysis revealed the immune infiltration in the tumor samples. With the heatmap, we could observe the estimated proportion of immune cells in the high and low FTRS groups (**Figure 8O**). From the box line plot, we could easily see that there was a statistically significant difference in immune cell expression between the different groups (**Figure 8P**). Also, we were able to find that the relatively high expression of immune cells in the high FTRS group was positively correlated with the risk score (**Figures 8Q, R**). For example, T cells regulatory (Treg) relatively high expression in the high FTRS group and positive correlation with risk score represented a poor prognosis. Tregs have been documented in malignant tumors, where they promote tumor progression by suppressing effective anti-tumor immunity and are associated with a poor prognosis in various types of human cancers (104). In addition, we used ESTIMATE to calculate the stromal score, immune score, and ESTIMATE score for the high FTRS group and low FTRS group, and found that all the scores of the high FTRS group were significantly higher than those of the low FTRS group, which indicated that the level of immune cell infiltration in the tumor samples of the high FTRS group was higher (**Figure 8S**). In addition, tumor purity was higher in the low FTRS group than in the high FTRS group (**Figure 8T**). Since low tumor purity was associated with extensive infiltration of stromal and immune cells, it was independently associated with shorter survival time and faster recurrence, and significantly correlated with mesenchymal, invasive, and metastatic phenotypes suggesting a poor prognosis that corresponds to the prognostic curve. Finally, TIDE is a tool used to comprehensively assess tumor immune escape mechanisms and predict response to immune checkpoint inhibitor (ICI) therapy, with higher TIDE scores correlating with poorer immune checkpoint inhibition therapy (105), and violin plot of high FTRS group scores being significantly greater than lower groups (**Figure 8U**).

### 3.9 Identification of differentially expressed genes and their enrichment analysis results and drug sensitivity analysis

**Figure 9:**
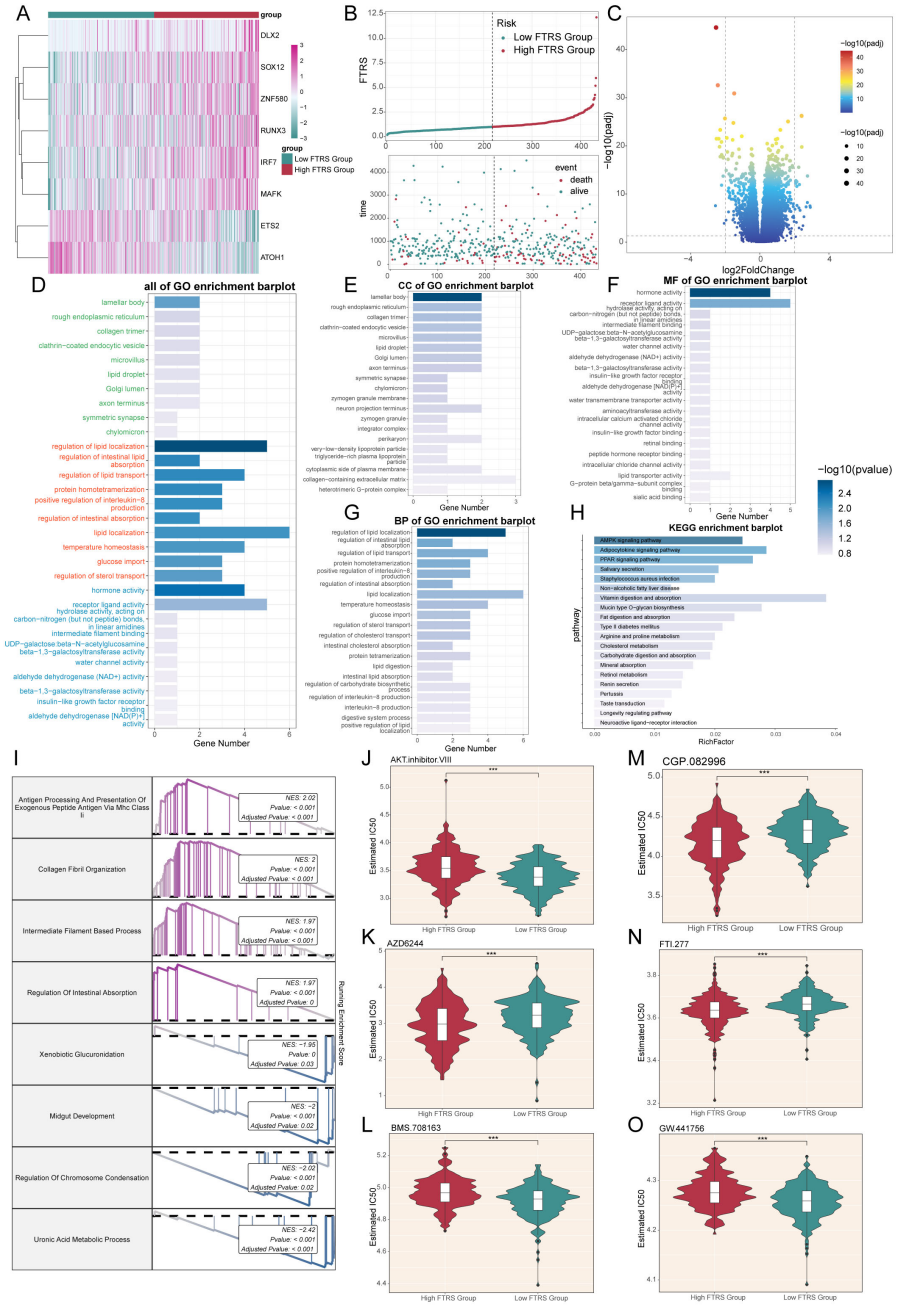
Enrichment analysis and drug sensitivity analysis in different risk groups. **(A)** Heatmap displayed differential expression of model genes, with color scale based on normalized data. **(B)** Curve chart illustrated the risk scores of high and low FTRS groups, and scatter plot depicted survival/death events over time for both groups. **(C)** Volcano plot showed significantly differentially expressed genes. Each spot represents a gene. **(D-H)** Bar charts separately presented the enrichment analysis results of differential genes in all of GO, GO-CC, GO-MF, GO-BP and KEGG pathways for high and low FTRS groups. **(I)** Results of GSEA of differentially expressed genes. **(J-O)** Violin plots demonstrated the sensitivity of tumor cells in the high and low FTRS risk groups to different drugs. *** represents P ≤ 0.001.

Immediately following the previous section, we proceeded to show the differential expression patterns of the genes used to construct the model using heatmap (**Figure 9A**). In addition, the curve and scatter plots showed the differences in risk scores and survival results between the two risk groups, suggesting that the high FTRS group was associated with a poorer prognosis, corresponding to the previous section (**Figure 9B**).

Next, volcano plot showed up- and down-regulated DEGs (**Figure 9C**). The filtering criteria for fold change were based on a log2 fold change threshold of 2, which meant that only genes with a fold change greater than or equal to 4 (or less than or equal to -4) were considered as differentially expressed. In addition to this fold change criterion, a p-value cutoff of 0.05 was applied to further refine the selection of significant DEGs. We then used these DEGs to gain insight into the biological processes involved by employing various enrichment methods. All GO enrichment results and their terms were shown, with different colors representing different subclasses (**Figure 9D**). GO-CC enrichment analysis showed that DEGs were predominantly enriched in lamellar body and rough endoplasmic reticulum, the latter of which suggested that there may be abundant protein synthesis in high-risk groups (**Figure 9E**). In GO-MF enrichment analysis, we observed enrichment in hormone and receptor ligand activity, suggesting that there may be an abundance of communication (**Figure 9F**). As for GO-BP enrichment analysis, the items mainly including regulation of lipid localization, intestinal lipid absorption and lipid transport (**Figure 9G**). All of the above enriched items are associated with lipid metabolism, and the former has been richly studied for its association with cancer. Dysregulated lipid metabolism is one of the most prominent metabolic alterations in cancer, and cancer cells utilize lipid metabolism for energy, biofilm components, and signaling molecules required for proliferation, survival, invasion, metastasis, and response to tumor microenvironmental influences and cancer therapy (106, 107). For another enrichment method analysis KEGG, the pathways were mainly AMPK, adipocytokine and PPAR signaling pathway (**Figure 9H**). Among them, AMPK plays a crucial role in maintaining energy homeostasis and metabolism, and its aberrant activation can lead to pro-carcinogenic effects, which is a double-edged sword (108); recent studies also show that AMPK signaling can also reversibly regulate highly active MAPK signaling in cancer cells by phosphorylating its key component, the RAF/KSR family of kinases, which not only affects carcinogenesis, but also the outcome of targeted cancer therapy against MAPK signaling. And there was an experiment showing that adipocytokines were involved in the carcinogenesis process (109), and an adipocytokine, LCN-2, has been reported to have the ability to destroy the extracellular matrix, which may lead to cancer progression and metastatic spread (110). At the same time, previous article had shown that PPAR-d upregulation increases susceptibility to colon tumorigenesis, which may impact the development of strategies to molecularly target PPAR-d in cancer and non-cancer diseases (111). Through the GSEA results we found that up-regulated DEGs were mainly enriched in the processes of antigen processing and presentation of exogenous peptide antigen, collagen fibril organization, intermediate filament-based process and regulation of intestinal absorption, while down-regulated DEGs were enriched in xenobiotic glucuronidation, midgut development, regulation of chromosome condensation and uronic acid metabolic process (**Figure 9I**).

Finally, in order to study the sensitivity of the high and low FTRS groups to different drugs, we predicted the drug sensitivity of each patient based on the drug sensitivity data in the GDSC database using the “pRRophetic” R software package. The results showed that the drugs AZD6244, CGP.082996, and FTI.277 had lower IC50 values and higher drug sensitivities in tumor cells of the high FTRS group, whereas the other drugs had lower IC50 values in tumor cells of the low FTRS group (**Figures 9J–O**).

### 3.10 *In vitro* experimental verifications of *DLX2*

**Figure 10:**
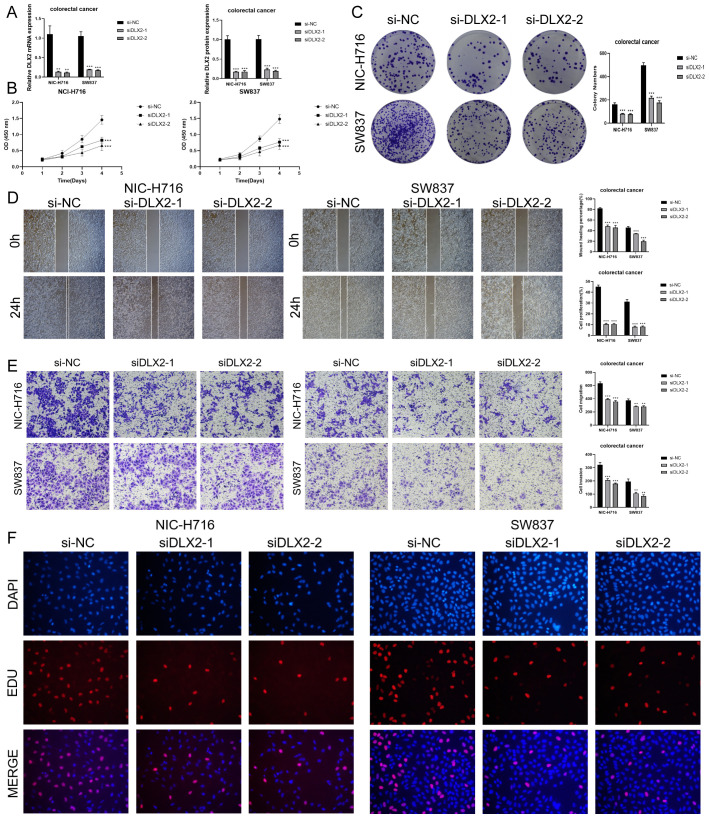
*In vitro* experiments confirmed the effects of *DLX2* knockdown. **(A)** *DLX2* knockdown significantly reduced the expression levels of mRNA and protein in both experimental groups. ** stands for P ≤ 0.01 and *** represents P ≤ 0.001. **(B)** CCK-8 assay showed that tumor cell viability was significantly decreased after *DLX2* knockdown compared with the control group. *** represents P ≤ 0.001. **(C)** Colony formation assays revealed a significant reduction in colony numbers after *DLX2* knockdown. *** represents P ≤ 0.001. **(D)** The scratch assay indicated that *DLX2* knockdown inhibited tumor cell migration. Bar graphs showed a significant reduction in both tumor cell migration and proliferation capabilities post-*DLX2* knockdown. *** represents P ≤ 0.001. **(E)** Transwell experiments indicated that *DLX2* knockdown inhibited the migration and invasion capabilities of tumor cells in the NIC-H716 and SW837 cell lines. ** stands for P ≤ 0.01 and *** represents P ≤ 0.001. **(F)** EDU staining assay confirmed that *DLX2* knockdown inhibited the proliferation of tumor cells by DAPI staining for comparison.

Through the previous construction of CRC prognostic model, we obtained the prognostically strongly related gene *DLX2*. To further investigate its role in CRC, we performed *in vitro* experiments using NCI-H716 and SW837 cell lines. First, we knocked down *DLX2* and measured mRNA and protein expression levels before and after knockdown. Compared with the control group, we observed a significant decrease in both mRNA and protein expression levels in both cell lines (**Figure 10A**). Subsequently, CCK-8 assay showed that tumor cell viability was significantly decreased after *DLX2* knockdown (**Figure 10B**). Colony formation assay confirmed that *DLX2* gene knockdown inhibited tumor cell aggregation (**Figure 10C**). In addition, after *DLX2* knockdown, the migratory and invasive abilities of tumor cells were assessed using the scratch and the transwell method, and the results showed that the levels of migration and invasion were significantly reduced (**Figures 10D, E**). Finally, EDU staining method also confirmed these results (**Figure 10F**). Together, these results indicated that knockdown of *DLX2* inhibited tumor cell activity, proliferation, migration, and invasion, thereby suppressing tumor growth.

## 4 Discussion

CRC is a highly heterogeneous disease with complex interactions between tumor cells and the surrounding microenvironment, making it difficult to fully understand the mechanisms driving progression and drug resistance (112–114). scRNA-seq has emerged as a powerful tool for revealing cellular diversity and gene expression profiles within tumors, allowing a more nuanced understanding of the cellular states and signaling pathways that contribute to CRC pathogenesis (115). Recent studies have shown that different tumor cell subtypes and the tumor microenvironment play a role in influencing CRC progression, immune evasion, and treatment response (116, 117). By utilizing scRNA-seq data, we can identify key cellular subtypes and regulatory networks that are critical for tumor growth, metastasis, and treatment response. This study builds on existing research by analyzing the cellular composition of CRC, identifying novel tumor cell subtypes, and mapping developmental trajectories and constructing prognostic models, ultimately providing new insights into the molecular and cellular dynamics of CRC.

First, we identified 8 distinct cell types, highlighting the complexity of the TME in CRC. Notably, as CRC progressed from normal to adenoma and carcinoma, the abundance of epithelial cells (EPCs), which have been considered a major driver of tumorigenesis due to their origin in the intestinal epithelium, increased markedly, and were transformed into tumor cells via EMT (118), an observation that is consistent with previous findings that epithelial expansion and transformation is associated with an increase in colorectal tumors with increased malignancy (119). The immunosuppressive nature of TME in CRC is highlighted by the progressive increase in proliferating cells and decrease in immune cells such as T and NK cells and B cells as the cancer progresses. The decrease in immunoreactivity at the stage of carcinogenesis suggests the presence of an underlying immune evasion mechanism, a phenomenon well documented in many types of solid tumors (120–122). This immunosuppression may also explain why immunotherapy has had limited success in patients with CRC, especially those without high microsatellite instability (MSI) (123). Subsequently, the identification of 7 TCs subtypes provided important information for understanding the cellular basis of CRC progression. The enrichment of C0 *FXYD5*+ TCs in cancer tissues and their association with poor prognosis confirmed previous studies that *FXYD5* expression is associated with increased epithelial-mesenchymal transition (EMT) and metastatic potential (89, 90). The different tissue distribution patterns of these subtypes reinforce the concept of CRC tumor heterogeneity. For example, C0 *FXYD5*+ TCs are abundantly present in cancerous tissues, whereas C2 *MUC2*+ TCs and C4 *OTOP2*+ TCs are predominantly present in normal and para-cancer tissues. This spatial and molecular heterogeneity highlights the need for a precision medicine approach to treating CRC, as different subtypes may respond differently to therapeutic interventions.

Studies of stemness genes in various tumor subtypes have revealed that *MYC* is significantly expressed in C0 *FXYD5*+ TCs, suggesting that this subtype has a high proliferative potential and is critical for tumor growth. Stemness has long been considered one of the hallmarks of cancer, and higher levels of stem-like features are associated with tumor aggressiveness and drug resistance (124–126). Pseudotime analyses using Monocle and Slingshot suggest that TCs follow distinct developmental trajectories. Lineage 1 appears to represent the key lineage associated with CRC tumorigenesis. Our findings also suggest that targeting key regulators on this trajectory, such as those driving the transition from C1 *APCDD1*+ to C0 *FXYD5*+ TCs, may provide novel strategies to prevent CRC progression. GSEA showed that C0 *FXYD5*+ TCs subtypes were highly involved in metabolic processes such as oxidative phosphorylation and aerobic respiration. This is consistent with the established concept of metabolic reprogramming in cancer cells, which enhances energy production through processes such as glycolysis and oxidative phosphorylation to support rapid cell proliferation (127–129). The enrichment of ribosome biogenesis and protein synthesis pathways in this subtype further emphasizes its role in maintaining the high metabolic demands of cancer cells (130). The up-regulation of genes such as *TMSB10, S100A10*, and *GSTP1* in the C0 subtype suggests that they are closely associated with the progression and poor prognosis of CRC. These genes are associated with tumor metastasis and chemotherapy resistance, and thus are potential targets for therapeutic intervention (94, 96, 97). SCENIC analysis identified several key TFs, including *ETV4, MYC*, and *XBP1*, which are major regulators of C0 *FXYD5*+ TCs subtypes. In particular, the role of *ETV4* in promoting CRC invasion and metastasis through activation of the EMT program highlights its potential as a therapeutic target (100).

In the end, our analysis highlights several key genes and pathways associated with poor CRC prognosis. A risk-scoring model based on the expression of eight genes, including *DLX2* and *SOX12*, provides a reliable framework for predicting the overall survival of CRC patients. Validation of this model in the TCGA cohort emphasizes its potential clinical utility in stratifying patients according to risk and adjusting treatment strategies accordingly. In addition, immune infiltration analysis showed that higher infiltration of regulatory T cells (Tregs) was associated with a poorer prognosis, which is consistent with previous findings that Tregs promote immune evasion in CRC by suppressing anti-tumor immune responses (104, 131, 132). Tumors with high expression of *FXYD5*+ TCs have higher immune cell infiltration and lower tumor purity, an observation that suggests that these tumors may be more resistant to immune checkpoint blockade therapy. This finding has important implications for the design of immunotherapies for the treatment of CRC, especially for high-risk patients with *FXYD5*+ TCs subtypes.

In conclusion, the present study describes in detail the cellular and molecular characteristics of CRC using scRNA-seq technology. The identification of different tumor subtypes, their developmental trajectories and key regulators provides new insights into the progression of CRC. The findings highlight potential prognostic markers and therapeutic targets, including *FXYD5*+ TCs, stem cell-associated genes, and key TFs, which could guide future interventions in CRC, and *in vitro* experiments were performed to validate the strongly prognostic-related gene *DLX2*. Given the complexity and heterogeneity of CRC, our study highlights the importance of a personalized approach in CRC treatment with the ultimate goal of improving patient prognosis.

However, our experiments still have shortcomings. First, although the study identified several key tumor cell subtypes and related markers, these findings were based only on bioinformatics analysis and *in vitro* experiments. Experimental validation, such as *in vivo* functional testing, is needed to confirm the role of these markers and subtypes in CRC progression and their potential as therapeutic targets. Second, although this study described immune cell types such as T and NK cells, B cells, and Tregs, there is limited understanding of the mechanisms of how these immune cells interact with tumor cells. A more detailed analysis of immune signaling pathways, immune cell function, and the potential for immunotherapeutic interventions could enhance the impact of this study, especially given the increasingly important role that immunotherapy is playing in the treatment of CRC. In addition, although the study identified several prognostic markers and developed a risk score model, it lacked sufficient correlation with clinical outcomes such as patient survival, response to treatment, or relapse rate. The inclusion of longitudinal clinical data would help validate the prognostic value of these markers and support their potential application in clinical practice. In future studies, we will address these limitations, improve the robustness of the findings, and expand their application in the diagnosis and treatment of CRC.

## Data Availability

The original contributions presented in the study are included in the article/**Supplementary Material**. Further inquiries can be directed to the corresponding authors.

## Supplementary material

Supplementary Figure 1 The heatmap demonstrated the relative expression intensity of each cell in each chromosomal region, the upper heatmap represents the demonstrated results of the reference cells (T and NK cells), and the lower heatmap represents the demonstrated results of the target cells (EPCs). Each row of the graph represents one cell and each column represents one gene. Red colour indicates CNV amplification, blue colour indicates CNV deletion, darker colour indicates more obvious CNV variation.
