# Supplementary figures and images for: Innovative strategies to optimise colorectal cancer immunotherapy through molecular mechanism insights
Source: Front Immunol. 2024 Dec 9;15:1509658. doi: 10.3389/fimmu.2024.1509658 (PMC11663906; doi:10.3389/fimmu.2024.1509658)

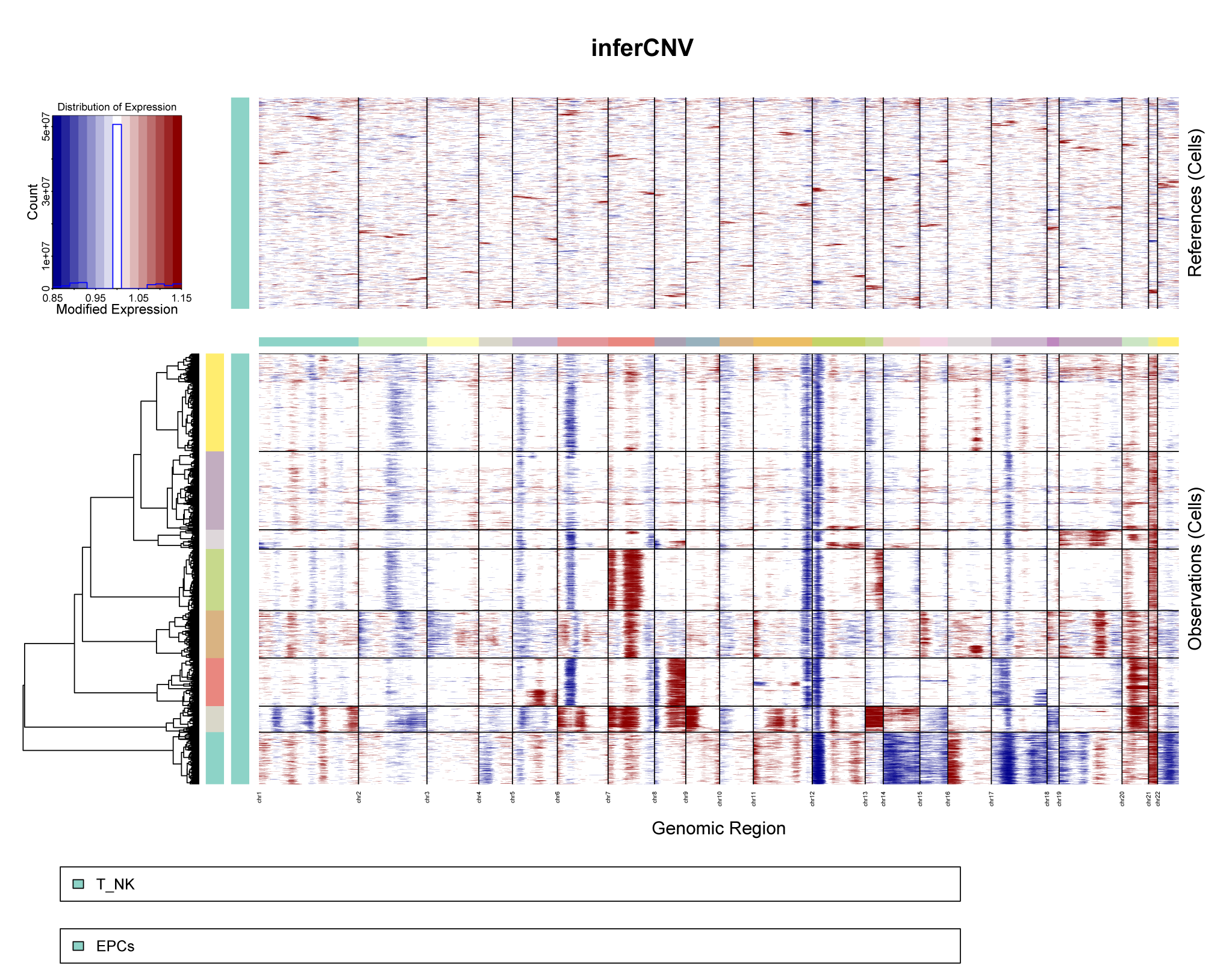

Supplement: Supplementary Figure 1 — The heatmap demonstrated the relative expression intensity of each cell in each chromosomal region, the upper heatmap represents the demonstrated results of the reference cells (T and NK cells), and the lower heatmap represents the demonstrated results of the target cells (EPCs). Each row of the graph represents one cell and each column represents one gene. Red colour indicates CNV amplification, blue colour indicates CNV deletion, darker colour indicates more obvious CNV variation. [file Image1.tif]
